# Risk factors for maternal mortality in a Tertiary Hospital in Kenya: a case control study

**DOI:** 10.1186/1471-2393-14-38

**Published:** 2014-01-22

**Authors:** Faith Yego, Catherine D’Este, Julie Byles, Jennifer Stewart Williams, Paul Nyongesa

**Affiliations:** 1 Department of Health Policy and Management, Moi University, P. O Box 4606, Eldoret 30100, Kenya; 2 University of Newcastle, HMRI Building, Callaghan, NSW 2308, Australia; 3 Centre for Clinical Epidemiology and Biostatistics, Callaghan, NSW 2308, Australia; 4 Research Centre for Gender, Health and Ageing, Callaghan, NSW 2308, Australia; 5 Department of Reproductive Health, Moi University, P.O. Box 4606, Eldoret 30100, Kenya; 6 National Centre for Epidemiology and Population Health, ANU College of Medicine, Biology and Environment, The Australian National University, Canberra, ACT, Australia; 7 Department of Public Health and Clinical Medicine Epidemiology and Global Health, Umeå University, Umeå, Sweden

**Keywords:** Maternal mortality, Tertiary hospital, Risk factors, Kenya

## Abstract

**Background:**

Maternal mortality is high in Africa, especially in Kenya where there is evidence of insufficient progress towards Millennium Development Goal (MDG) Five, which is to reduce the global maternal mortality rate by three quarters and provide universal access to reproductive health by 2015. This study aims to identify risk factors associated with maternal mortality in a tertiary level hospital in Kenya.

**Methods:**

A manual review of records for 150 maternal deaths (cases) and 300 controls was undertaken using a standard audit form. The sample included pregnant women aged 15-49 years admitted to the Obstetric and Gynaecological wards at the Moi Teaching and Referral Hospital (MTRH) in Kenya from January 2004 and March 2011. Logistic regression analysis was used to assess risk factors for maternal mortality.

**Results:**

Factors significantly associated with maternal mortality included: having no education relative to secondary education (OR 3.3, 95% CI 1.1-10.4, p = 0.0284), history of underlying medical conditions (OR 3.9, 95% CI 1.7-9.2, p = 0.0016), doctor attendance at birth (OR 4.6, 95% CI 2.1-10.1, p = 0.0001), having no antenatal visits (OR 4.1, 95% CI 1.6-10.4, p = 0.0007), being admitted with eclampsia (OR 10.9, 95% CI 3.7-31.9, p < 0.0001), being admitted with comorbidities (OR 9.0, 95% CI 4.2-19.3, p < 0.0001), having an elevated pulse on admission (OR 10.7, 95% CI 2.7-43.4, p = 0.0002), and being referred to MTRH (OR 2.1, 95% CI 1.0-4.3, p = 0.0459).

**Conclusions:**

Antenatal care and maternal education are important risk factors for maternal mortality, even after adjusting for comorbidities and complications. Antenatal visits can provide opportunities for detecting risk factors for eclampsia, and other underlying illnesses but the visits need to be frequent and timely. Education enables access to information and helps empower women and their spouses to make appropriate decisions during pregnancy.

## Background

The maternal mortality ratio (MMR) is defined as “the ratio of the number of maternal deaths during a given period per 100,000 live births during the same time-period”. The global MMR is 210 per 100,000 live births [1]. Despite worldwide declines since 1990, the MMR is 15 times higher in developing than developed regions [1]. Sub-Saharan Africa has the highest MMR at 500 per 100,000 live births. In developed regions the MMR is 16 per 100,000 live births [1]. The target for Millennium Development Goal (MDG) Five is to reduce the global MMR by three quarters and to achieve universal access to reproductive health by 2015 [2]. In Kenya, the MMR has remained at 400-600 per 100,000 live births over the past decade - resulting in little or no progress being made towards achieving MDG Five [1,3].

The main direct causes of maternal death in developing countries include haemorrhage, sepsis, obstructed labour and hypertensive disorders [4]. The risk of death from haemorrhage is one in 1,000 deliveries in developing countries, compared with one in 100,000 in developed countries, and accounts for one third of the maternal deaths in Africa [5]. A study in Canada found increased risk of eclampsia among women with existing heart disease and anaemia [6]. A retrospective study undertaken at a tertiary hospital in Nigeria in 2007 found that the most common risk factors for maternal mortality were primaparity, haemorrhage, anaemia, eclampsia and malaria [7]. Risk factors for complications arising from infections include birthing under unhygienic conditions, poor nutrition, anaemia, caesarean section, membrane rupture, prolonged labour, retained products and haemorrhage [8].

In developing countries, indirect causes of maternal death include both previously existing diseases and diseases that develop during pregnancy. These include HIV, malaria, tuberculosis, diabetes, and cardiovascular disease, all of which and have an enormous impact on maternal and fetal outcomes during pregnancy [4].

Many individual and socioeconomic factors have been associated with high maternal mortality. These include lack of education, parity, previous obstetric history, employment, socioeconomic status, and types of care seeking behaviours during pregnancy. There is also evidence of increased risk of death among women who are less than 24 and older than 35 years [9]. A study in Tanzania found that low level of spouse education was a risk factor for maternal mortality [10]. Lack of knowledge regarding the need for skilled attendants is a barrier to women seeking care, especially during birth emergencies. A survey conducted in Kenya in 2006 showed that 15% of pregnant women were not informed of the importance of hospital deliveries [11]. In Nigeria, a cross-sectional survey revealed that the most common risk factors for maternal death were primigravidity (19%), and unbooked status (19%) [12]. Poverty has also been associated with adverse maternal outcomes, not directly, but as a contributor to maternal ability to access and utilise care where complications occur [13,14]. There is also evidence that contraceptive use is efficient for the primary prevention of maternal mortality in developing countries by about 44% [15].

Antenatal care (ANC) is very important during pregnancy. International organizations recommend a minimum of four visits, the administration of two doses of tetanus toxoid and folic acid supplementation during ANC attendance [16]. When women receive good care during the pre-partum period, they have been shown to be at less risk of maternal morbidity and mortality, since they had a higher likelihood of using a professional health facility during birth [10,17].

In the Kenya Demographic and Health Survey (2008-2009), it was reported that 92% of women received ANC from a skilled provider (doctor, nurse, or midwife), especially those who were more educated and resided in urban areas [3]. The report further showed that 83% of women who visited public hospitals were required to pay for antenatal services, which may explain why only 47% of antenatal women attended the recommended four visits [3]. Women had also been required to pay for delivery services until June 2013, when the Kenyan government rolled out a program where pregnant women can receive free maternity services in public hospitals.

Health systems functioning with adequate equipment, resources and trained personnel to handle maternal complications can reduce the risks of mortality. In Africa maternal deaths are associated with delayed referrals for women from lower level facilities, and where referral systems are not well equipped to handle emergency obstetric care [18]. The presence of skilled attendants during birth is also important in managing life threatening complications. In Kenya, the use of skilled attendants at delivery is currently 50% [19].

The Delay Model by McCarthy and Maine is a conceptual framework that has been used to assess factors contributing to maternal mortality in developing countries [20]. This framework attributes mortality to certain determinants that contribute to the delay in deciding to seek care, the delay in reaching a health facility, and the delay in receiving quality care upon reaching a health facility. In Kenya there has been insufficient progress made towards achieving MDG Five. The aim of this study is to identify risk factors associated with maternal mortality in a tertiary level hospital in Kenya. Using a framework adapted from the Delay Model, this study analyses four sets of determinants: individual and socio-demographic, maternal history, reproductive or obstetric, and hospital admission/health system.

## Methods

An unmatched case control study of women who delivered between January 2004 and March 2011 was conducted at Moi Teaching and Referral Hospital (MTRH) located in the Western region of the Rift Valley Province, Kenya [21]. As the second largest national hospital in Kenya with over 800 beds, MTRH provides a range of curative, preventive and rehabilitative health services to a population of about 400,000 inhabitants, and an indigent referral population of 16 million from Northern and Western Kenya [21]. The Mother and Baby Unit at MTRH at has an antenatal ward, post natal ward, labour ward, Newborn Unit (NBU) and two theatres dedicated for obstetrics. The bed capacity is approximately 20 for the antenatal and labour wards, and 50 for the post natal wards [21].

Cases (n = 150) were maternal deaths identified from a manual review of hospital records. Two controls (n = 300) were selected per case. Controls were surviving women who were admitted immediately preceding and following cases. Cases were selected retrospectively and sequentially from the most recent delivery until the required sample size was achieved. Trained staff collected information using a standard audit form. Abortion related deaths were excluded from the study.

Maternal hospital death was the outcome. This was a clearly defined adverse event certified by medical personnel. The data collection form included: mother’s age, mother’s marital status, mother’s education, spouse’s education, mother’s occupation, spouse’s occupation, and the source of funding for the delivery. Information relating to the mother’s medical history included: smoking, alcohol use, contraceptive use, previous abortion, previous twins, gravida, and pre-existing medical conditions. Obstetric or reproductive factors were pregnancy stage, labour stage, number of ANC visits, and place of ANC care. Health system factors included mode of delivery, qualification of birth attendant, and referral from another facility (yes/no). Information on the mother’s admission factors comprised: clinical cause of death or diagnosis on admission (e.g. eclampsia, dystocia haemorrhage, or comorbid causes), diastolic blood pressure (millimetres of mercury/mm Hg), systolic blood pressure (mm HG), haemoglobin level (grams per decilitre g/dL), pulse rate (beats per minute/bpm), and temperature (degrees Celsius/°C). The primary obstetric cause of death was that documented in the patient hospital and post mortem records.

### Statistical analyses

Analyses were performed using Stata version 10.0 (Stata-Corp, College Station, TX, USA). Following initial data checking and exploratory analysis, univariable logistic regression analysis was conducted for each potential risk factor. The multivariable models initially included all variables with p < 0.2 in the univariable models. Backward stepwise multiple logistic regression was undertaken separately for the four groups of risk factors in the framework adapted from the Delay Model, (individual and socio-demographic; maternal history; reproductive or obstetric; and admission). Variables were removed from the models where p-values > = 0.1 on the Likelihood Ratio Test. The variables in each of the final models were then included in a combined model and removed where p-values > = 0.1 in order to derive a final parsimonious model. Odds ratios (ORs), 95% confidence intervals and p-values are reported for all models. The reference group was the category with the lowest expected risk of death, or if there were few cases in this category, the group with the majority of respondents.

Assuming the probability of exposure in controls was 40% and the ratio of cases to controls was 1:2, with 80% power and a 5% level of significance, a sample of approximately 450 women (150 cases and 300 controls) was needed to detect an odds ratio of approximately 0.5 or 1.8.

Ethical approval was sought from the Human Research Ethics Committee (HREC) at the University of Newcastle and The Institute for Research and Ethics Committee (IREC) in Kenya.

## Results

Table 1 shows the demographic factors associated with maternal mortality. In this model, mother’s age and mother’s education were significantly associated with mortality. Relative to controls, cases had three times the odds of being aged 35-45 years rather than 15-24 years (OR 3.1, 95% CI 1.5- 6.2; p < 0.0001). Cases had eight times the odds of having no education versus secondary education compared with controls (OR 8.0, 95% CI 4.0-16.3; p < 0.0001).

**Table 1:** Individual and Socio-demographic risk factors for maternal mortality in a tertiary hospital in Kenya from January 2004 to March 2011

**Individual and socio-demographic characteristics**					
**Risk factor**	**Cases n = 150 n (%)**	**Controls n = 300n (%)**	**Unadjusted OR (95% CI)**	**Adjusted OR (95% CI)**^**a**^	**P-value**^**†**^
**Age**					<0.0001
15–24 years	66(44)	147(49)	1	1	
25–34 years	54(36)	129(43)	1.1(0.7–1.6)	1.4(0.9–2.4)	
35–45 years	30(20)	24(8)	3.0(1.6–5.6)	3.1(1.5–6.2)	
**Marital status**					
Married	111(74)	240(80)	1		
Single	39(26)	60(20)	1.4(0.9–2.2)		
**Mother’s education**					<0.0001
None	31(23)	17(6)	8.5(4.3–17.0)	8.0(4.0–16.3)	
Primary	66(50)	108(37)	2.9(1.8–4.6)	2.9(1.8–4.7)	
Secondary	36(27)	168(57)	1	1	
Spouse’s education*					
None	20(22)	14(6)	5.9(2.8–12.7)		
Primary	30(33)	46(20)	2.7(1.5–4.8)		
Secondary	40(44)	166(74)	1		
**Occupation of mother**					
Unemployed	106(71)	176(59)	1		
Formal employment	28(19)	88(29)	0.5(0.3–0.9)		
Informal employment	15(10)	35(12)	0.7(0.4–1.4)		
**Occupation of spouse**					
Informal employment	59(41)	124(42)	1		
Formal employment	47(32)	114(38)	0.9 (0.5–1.3)		
NA	39(27)	60(20)	1.4(0.8–2.3)		
**Funding for delivery**					
Self	105(70)	210(70)	1		
Insurance	16(11)	47(16)	0.7(0.4–1.3)		
Waiver	28(19)	43(14)	1.3(0.8–2.2)		

*Spouse’s education was not included in the multiple regression model due to high cases of missing data and correlation with mothers education.

^a^Adjusted for variables included in the final demographic model.

^†^P- value for Likelihood Ratio Test in the adjusted model.

Reference category for logistic regression represented by 1.

Numbers may not add to total sample due to missing values.

Table 2 shows the association between maternal history of prevailing conditions and obstetric and reproductive factors with maternal mortality. After adjusting for all other factors in the model, cases had higher odds than controls of having a history of maternal alcohol use (OR 2.5, 95% CI 1.2-5.3; p = 0.018), more than five previous pregnancies (OR 2.6, 95% CI 1.4-4.8; p = 0.0049), and a history of pre-existing illnesses (OR 3.0, 95% CI 1.7-5.3; p < 0.0001). Contraceptive use was protective (OR 0.3 95% CI 0.1-0.6; p = 0.0007). Table 2 also shows obstetric and reproductive characteristics associated maternal mortality. Compared to controls, cases had higher odds of assisted or caesarean deliveries (OR 3.0 95% CI 1.5-5.6; p < 0.0006). Cases had higher odds than controls of having a doctor, rather than a nurse or midwife attend the birth (OR 4.1 95% CI 2.2-7.6; p < 0.0001). Relative to controls, cases had almost nine times the odds of arriving at hospital at the puerperium stage (OR 8.9, 95% CI 3.5, 22.7; p < 0.0001), and almost six times the odds of lack of antenatal care (OR 5.7, 95% CI 2.6-12.4; p < 0.0001).

**Table 2:** Mother’s history of prevailing conditions and obstetric characteristics associated with maternal mortality in a tertiary hospital in Kenya from January 2004 to March 2011

**History of prevailing medical conditions**					
**Risk factor**	**Cases n = 150 n (%)**	**Controls n = 300 n (%)**	**Unadjusted OR (95% CI)**	**Adjusted OR (95% CI)**^**a**^	**P-value**^**†**^
**Smoking**					
Yes	2(1)	7(2)	0.6(0.1–2.7)		
No	148(99)	286(98)	1		
**Alcohol**					0.018
Yes	16(11)	20(7)	1.6(0.8–3.2)	2.5(1.2–5.3)	
No	134(89)	273(93)	1		
**Contraceptives**					0.0007
Yes	17(11)	74(25)	0.4(0.2–0.7)	0.3(0.1–0.6)	
No	133(89)	220(75)	1		
**Abortion**					
Yes	11(7)	20(7)	1.1(0.5–2.4)		
No	139(93)	279(93)	1		
**Twins**					
Yes	10(7)	17(6)	1.2(0.5–2.7)		
No	140(93)	283(94)	1		
**Gravida**					0.0049
Primigravida	34(23)	104(35)	1		
Multigravida	73(49)	153(51)	1.5(0.9–2.4)	1.5(0.9–2.5)	
Grandmultigravida	43(29)	43(14)	3.1(1.7–5.4)	2.6(1.4–4.8)	
Underlying medical conditions*					
Yes	41(27)	29(10)	3.5(2.1–5.9)	3.0(1.7–5.3)	
No	109(73)	271(90)	1		
**Obstetric and reproductive characteristics**					
**Mode of delivery**					0.0006
Normal	64(43)	230(77)	1		
Assisted	27(18)	13(4)	7.5(3.6–15.3)	4.4(1.7–11.2)	
Caesarean	51(34)	57(19)	3.2(2.0–5.1)	3.0(1.5–5.6)	
Did not deliver	8(5)	0	omitted	omitted	
**Birth attendant**					<0.0001
Nurse/midwife	70(53)	264(89)	1	1	
Doctor	61(47)	31(11)	7.4(4.5–12.3)	4.1(2.2–7.6)	
**Pregnancy stage**					<0.0001
Intrapartum	61(42)	259(86)	1	1	
Antepartum	53(37)	29(10)	7.8(4.6–13.2)	3.0(1.5–6.2)	
Puerperium	30(21)	12(4)	10.6(5.1–21.9)	8.9(3.5–22.7)	
**Labour stage**					
Latent	42(42)	68(24)	1		
Active	37(37)	180(63)	0.3(0.2–0.6)		
Second stage	20(20)	34(12)	1.0(0.5–1.9)		
**Number of ANC visits**					<0.0001
1 to 3	71(50)	199(67)	1	1	
None	59(42)	14(5)	11.8(6.2–22.5)	5.7(2.6–12.4)	
Above 4	11(8)	83(28)	0.4(0.2–0.7)	0.6(0.3–1.2)	
**Place of ANC attendance**^**††**^					
Health centre	41(28)	122(41)	1		
Hospital	26(18)	60(20)	1.3(0.7–2.3)		
MTRH	19(13)	100(34)	0.6(0.3–1.0)		
None	59(41)	14(5)	12.5(6.3–24.8)		

*These include: HIV, malaria, rheumatic heart disease, cardiovascular disease, and diabetes.

^†^P- value for Likelihood Ratio Test in the adjusted model.

^a^Adjusted for variables included in the final demographic model.

Reference category for logistic regression represented by 1.

^††^Did not include ANC place because of high correlation with number of ANC visits.

Numbers may not add to total sample due to missing values.

Table 3 shows maternal admission factors associated with mortality. Admission from comorbid complications (OR 6.7, 95% CI 3.8-11.8; p < 0.0001), eclampsia (OR 4.7, 95% CI 1.6, 13.7; p = 0.0038), non-normal blood pressure (OR 7.5, 95% CI 1.5-37.7; p = 0.0039), tachycardia (OR 16.5, 95% CI 4.8-57.3; p < 0.0001), and being referred to MTRH (OR 3.3, 95% CI 1.9-5.7; p < 0.0001) were all statistically significant risk factors for maternal mortality.

**Table 3:** Maternal admission factors associated with maternal mortality in a tertiary hospital in Kenya from January 2004 to March 2011

**Admission factors**					
**Risk factor**	**Cases n = 150 n (%)**	**Controls n = 300 n (%)**	**Unadjusted OR (95% CI)**	**Adjusted OR (95% CI)**^**a**^	**P-value**^**††**^
**Comorbid complications**					<0.0001
Yes	93(62)	63(21)	0.2(0.1–0.3)	6.7(3.8–11.8)	
No	57(38)	237(79)	1	1	
**Eclampsia**					0.0038
Yes	33(22)	16(5)	5.0(2.7–9.4)	4.7(1.6–13.7)	
No	117(78)	284(95)	1	1	
**Dystocia**					
Yes	21(14)	51(17)	0.8(0.5–1.4)		
No	129(86)	249(83)	1		
**Haemorrhage**					
Yes	20(13)	15(5)	2.9(1.5–5.9)		
No	120(87)	285(95)	1		
Diastolic blood pressure (mm HG)**					0.0039
Normal	102(72)	280(96)	1	1	
Low	16(11)	2(1)	22.0(5.0–97.2)	7.5(1.5–37.7)	
High	24(17)	10(3)	6.6(3.0–14.3)	3.2(0.9–10.6)	
Systolic blood pressure (mm HG)**					
Normal	93(65)	271(93)	1		
Low	33(23)	6(2)	16.0(6.5–39.5)		
High	17(12)	15(5)	3.3(1.6–6.9)		
**Haemoglobin**^**†**^					
<10 g/dL	63(52)	43(20)	4.2(2.6–6.9)		
> = 10 g/dL	59(48)	171(80)	1		
**Pulse**					<0.0001
<110 bpm	103(71)	283(99)	1	1	
> = 110 bpm	41(28)	4(1)	28.2(9.8–80.6)	16.5(4.8–57.3)	
**Temperature**^**†**^					
<37.5°C	110(82)	205(95)	1		
> = 37.5°C	24(18)	11(5)	4.1(1.9–8.6)		
**Referral**					<0.0001
No	63(42)	234(78)	1	1	
Yes	87(58)	66(22)	4.9(3.2–7.5)	3.3(1.9–5.7)	

**Diastolic blood pressure was used as a proxy for systolic because of high correlation.

^†^Temp and haemoglobin were omitted in the adjusted model because of too many missing values.

^††^P- value for Likelihood Ratio Test in the adjusted model.

^a^Adjusted for variables included in the final demographic model.

Reference category for logistic regression represented by 1.

Numbers may not add to total sample due to missing values.

Table 4 shows the multivariable analysis combining all factors from the previous models. Statistically significant risk factors for maternal mortality included: no education, relative to secondary education (OR 3.3, 95% CI 1.1-10.4, p = 0.0284), history of pre-existing medical conditions (OR 3.9, 95% CI 1.7-9.2, p = 0.0016), doctor attendance at birth (OR 4.6, 95% CI 2.1-10.1, p = 0.0001), having no antenatal visits (OR 4.1, 95% CI 1.6-10.4, p = 0.0007), being admitted with eclampsia (OR 10.9 95% CI 3.7-31.9, p < 0.0001), having comorbid complications on admission (OR 9.0, 95% CI 4.2-19.3, p < 0.0001), having elevated pulse (OR 10.7, 95% CI 2.7-43.4, p = 0.0002), and being referred to MTRH (OR 2.1, 95% CI 1.0-4.3, p = 0.0459).

**Table 4:** Multivariable model showing risk factors for maternal mortality in a tertiary hospital in Kenya from January 2004 to March 2011

**Final multivariable model**				
**Risk factor**	**Cases n = 150 n (%)**	**Controls n = 300 n (%)**	**OR (95% CI)**	**Likelihood ratio test X**^**2 **^**(df), p-value**
**Mothers education**				
Secondary	36(27)	168(57)	1	
None	31(23)	17(6)	3.3(1.1–10.4)	
Primary	66(50)	108(37)	2.4(1.1–5.3)	7.13(2), 0.0284
**Underlying medical conditions**				
No	109(73)	271(90)	1	
Yes	41(27)	29(10)	3.9(1.7–9.2)	9.95(1), 0.0016
**Birth attendant**				
Nurse/midwife	70(53)	264(89)	1	
Doctor	61(47)	31(11)	4.6(2.1–10.1)	14.41(1), 0.0001
**Number of ANC visits**				
1 to 3	71(50)	199(67)	1	
None	59(42)	14(5)	4.1(1.6–10.4)	
Above 4	11(8)	83(28)	0.5(0.2–1.2)	14.56(2), 0.0007
**Comorbid complications**^**†**^				
No	57(38)	237(79)	1	
Yes	93(62)	63(21)	9.0(4.2–19.3)	36.33(1), <0.0001
**Eclampsia**				
No	117(78)	284(95)	1	
Yes	33(22)	16(5)	10.9(3.7–31.9)	21.29(1), <0.0001
**Pulse**				
<110 bpm	103(71)	283(99)	1	
> = 110 bpm	41(28)	4(1)	10.7(2.7–43.4)	13.58(1), 0.0002
**Referral**				
No	63(42)	234(78)	1	
Yes	87(58)	66(22)	2.1(1.0–4.3)	3.98(1), 0.0459

Final model included all variables with P-value of 0.1 or less on the likelihood ratio test.

Reference category for logistic regression represented by 1.

^†^These include: HIV, malaria, rheumatic heart disease, cardiovascular disease, and diabetes.

Numbers may not add to total sample due to missing values; the final model included 367 observations.

## Discussion

In the multivariable analysis of each of the four groups of risk factors (socio-demographic, maternal history, reproductive/ obstetric and admission factors) variables significantly associated with maternal mortality included: age, education, alcohol use, contraceptive use, gravida, pre-existing medical conditions, mode of delivery, type of birth attendant, pregnancy stage, number of ANC visits, having comorbid complications on admission, eclampsia, diastolic blood pressure, elevated pulse, and referral status. However, in the final model combining only significant factors from the four separate sets of analyses into a parsimonious model, only education, underlying medical conditions, birth attendant, number of ANC visits, having comorbid complications, eclampsia, having an elevated pulse on admission, and referral status were significant risk factors for maternal mortality.

Cases had three times the odds of having no education versus secondary education compared with controls. This is in agreement with another study that also reported a higher risk of mortality among illiterate women [22]. This finding is important since it emphasizes the role of education for both the mother and her spouse in obtaining and understanding the benefits of good health and being able to make appropriate decisions during pregnancy. It is important to note that despite the woman’s weaker role in decision-making in African settings, education has a strong influence on mortality. In this study, we used mother’s education as a proxy for the husband’s education. Although there was considerable missing data for spouse’s education, there was correlation between these two education variables.

Having no antenatal care during pregnancy was associated with mortality in this study, a finding which corresponds with those of other studies [22,23]. Antenatal care is important in screening for pre-existing illnesses and complications in the early stages of pregnancy that could impact adversely during pregnancy and childbirth [24]. Since ANC coverage is high in Kenya, there is a need to scale up interventions that empower women to make at least four visits during pregnancy as recommended by international organizations [16,19].

The findings here were that comorbid conditions including HIV, malaria, rheumatic heart disease, cardiovascular disease, and diabetes contributed to maternal deaths. This is contrary to other research showing that direct pregnancy complications are the leading causes of maternal deaths [4]. However, other research shows that the significant increase in MMRs in Sub-Saharan Africa are predominantly due to increasing HIV prevalence in that region [25]. The finding that the odds of comorbid conditions were higher in cases than controls also demonstrates the importance of ANC for screening, detection and management of underlying illnesses that could potentially pose a threat to the mother during pregnancy and childbirth.

Contraceptive use was protective for maternal mortality, which coincides with findings from another study that found that maternal mortality would be 77% higher globally in the absence of family planning programs and contraceptive use [15]. The role of contraceptives in tackling maternal mortality has been through reducing exposure to incidence of pregnancy, lowering hazards of fragility from high parity pregnancies, reducing vulnerability to abortion risks, and postponing pregnancies, especially in countries with high fertility rates [15].

In this study, hypertensive disorders during pregnancy were higher among cases than controls. Our study demonstrated increased odds of eclampsia in cases, which is in agreement with another study that found that the delay in diagnosis, triage, transport and treatment of eclampsia increases the risk of maternal death [26]. There is evidence that screening for hypertensive conditions during the antenatal period plays a significant role in reducing the risk of death to the mother [13]. This study also found higher odds of elevated pulse amongst cases, which could explain the increased risk of death due to eclampsia. After adjusting for other factors, haemorrhage was not significantly associated with mortality possibly as a result of hospital protocols for management of haemorrhage.

This study found health care system related factors that identified cases as being at risk including doctor attendance at birth and referrals. Cases had higher odds than controls of a doctor attending their delivery, potentially because they were diagnosed with the most difficult complications. This has been previously reported, especially in low resource settings where uptake of professional birth attendants is low hence women only seek help when the condition is critical or too late for the doctor to save their lives [4]. Cases had twice the odds of referral relative to controls, potentially because the number of referrals represented over half of the cases who were referred following complications of birth.

This study provides information that is important for the identification of risk factors that contribute to maternal mortality in the second largest referral hospital in Kenya. It also provides information that will aid in identifying areas of improving health facilities locally and nationally in terms of referrals, antenatal care, and the availability of skilled birth attendants who are able to manage pregnancy related complications. This study is timely given the free maternity program roll out in Kenya since June 2013. Importantly, these findings will inform policy makers about ways of strengthening the health system and promoting more hospital births.

This study has some limitations. Firstly, it only includes deaths that occurred during the hospital admission and therefore the risk factors identified here were specifically associated with in-hospital mortality. Pregnancy related mortality that occurs outside hospital may have other risk factors that were not identified here. Secondly, bias may have resulted from the misclassification of causes of death data and missing information in some fields.

## Conclusions

This study highlights risk factors for mortality at a tertiary hospital in Kenya showing the importance of antenatal care and maternal education in preventing maternal mortality. The findings are timely given Kenya’s limited progress towards achieving MDG Five by 2015. Antenatal visits provide opportunities for the detection of risk factors for eclampsia and other underlying illnesses that may put a mother at risk during birth. There is need to focus on integrated care throughout the pregnancy by improving women’s knowledge and empowering them to take an active role in their own health as well as gaining access to skilled care at birth and during pregnancy.

## Abbreviations

MDG: Millennium development goal; MMR: Maternal mortality ratio; ANC: Antenatal care; MTRH: Moi teaching and referral hospital; NBU: Newborn unit; WHO: World Health Organization; HIV: Human immunodeficiency virus.

## Competing interests

The authors declare that they have no competing interests.

## Authors’ contributions

FY conceived the study. FY, CD, JB, JSW, and PN all contributed to the protocol design, questionnaire design and ethics application process. FY contributed in data collection and extraction. PN contributed in providing consultation and advice during data extraction. FY, CD, PN and JB contributed to data analysis and writing of the paper. CD, JB, FY and JSW contributed to the drafting and editing the paper. All authors contributed to reviewing the paper and approved the final version for publication.

## Pre-publication history

The pre-publication history for this paper can be accessed here:

http://www.biomedcentral.com/1471-2393/14/38/prepub
